# Surface Modification of Dental Titanium Implant by Layer-by-Layer Electrostatic Self-Assembly

**DOI:** 10.3389/fphys.2017.00574

**Published:** 2017-08-07

**Authors:** Quan Shi, Zhiyong Qian, Donghua Liu, Hongchen Liu

**Affiliations:** ^1^Department of Stomatology, Chinese PLA General Hospital Beijing, China; ^2^School of Biological Science and Medical Engineering, Beihang University Beijing, China; ^3^Department of Advanced Interdisciplinary Studies, Institute of Basic Medical Sciences and Tissue Engineering Research Center, Academy of Military Medical Sciences (AMMS) Beijing, China

**Keywords:** dental implant, layer-by-layer self-assembly, titanium, surface modification, osseointegration, soft tissues healing, antibacterial properties

## Abstract

*In vivo* implants that are composed of titanium and titanium alloys as raw materials are widely used in the fields of biology and medicine. In the field of dental medicine, titanium is considered to be an ideal dental implant material. Good osseointegration and soft tissue closure are the foundation for the success of dental implants. Therefore, the enhancement of the osseointegration and antibacterial abilities of titanium and its alloys has been the focus of much research. With its many advantages, layer-by-layer (LbL) assembly is a self-assembly technique that is used to develop multilayer films based on complementary interactions between differently charged polyelectrolytes. The LbL approach provides new methods and applications for the surface modification of dental titanium implant. In this review, the application of the LbL technique to surface modification of titanium including promoting osteogenesis and osseointegration, promoting the formation and healing of soft tissues, improving the antibacterial properties of titanium implant, achieving local drug delivery and sustained release is summarized.

## Introduction

Titanium and its alloys exhibit good biocompatibility, mechanical properties and machinability, and implants composed of titanium and titanium alloys as raw materials are widely used in the fields of biology and medicine (Neoh et al., 2012; Lemons, 2016). In oral medicine, titanium is the most ideal and the most commonly used dental implant material. Currently, dental implants are used in increasing numbers of patients with dentition defects or edentulous patients due to its good and comfortable restorative effects (Shi et al., 2016).

The improvement of the success rate of dental implants, shorten the treatment time, reduce the occurrence of peri-implantitis and peri-implant mucositis remains an important area of research with regards to oral implantology. An increasing number of studies have been devoted to modifying the surface of titanium and titanium alloy to increase their biological activity and promote osseointegration and soft tissue healing. In addition, the promotion of the osseointegration of titanium dental implants in diabetic and osteoporosis patients via the surface modification of the titanium implants must be addressed (Alghamdi et al., 2014).

To achieve these objectives, researchers have investigated a variety of methods (Jemat et al., 2015; Łępicka and Grądzka-Dahlke, 2016; Zemtsova et al., 2016), including blasting, etching, etc. (Supplementary Table 1). Besides, many substances are applied to titanium surfaces as a coating, including bone morphogenetic protein (BMP), chitosan and anatase, etc., by which to achieve early osseointegration, ensure a long-term bone-to-implant contact (BIC) and improve antibacterial effect. The layer-by-layer (LbL) electrostatic self-assembly technique proposed by Decher (1997) has attracted extensive attention because it provides a simple, useful and versatile methodology for material surface modifications.

LbL assembly is a versatile self-assembly technique that has been used to formulate polyelectrolyte multilayers (PEMs) using the electrostatic attractions between the assembled components. Recent studies of LbL assembly have demonstrated its utility in a wide range of applications including energy storage (Xiang et al., 2012), tissue and cell engineering (Shukla and Almeida, 2014), and drug delivery (Choi and Hong, 2014). In addition, the LbL technique has recently been more widely used for surface modification of dental titanium implant. Therefore, we have reviewed the progress of the application of the LbL technique for the surface modification of titanium and its alloys.

## Principle and advantages of LbL technique

### Principle of LbL technique

**Figure 1:**
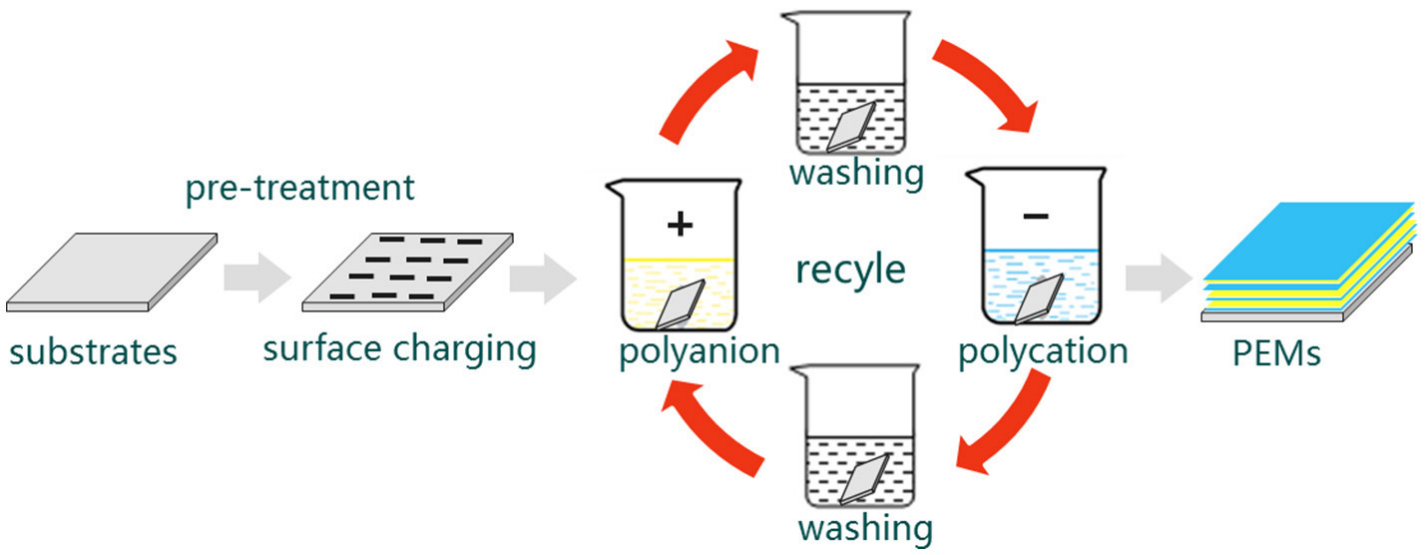
A schematic illustration of LbL self-assembly process. First, a charged substrate is immersed in a solution consisting of oppositely charged polyelectrolytes to form the first monolayer via absorption. Then, followed by a washing step to remove the weakly bound or unbound species and prevent cross contamination of oppositely charged polyelectrolytes. After that, the monolayer-coated substrate is immersed in another solution of oppositely charged polyelectrolytes to form the second monolayer via absorption. This process is repeated until the desired multilayers are formed. PEMs: formulate polyelectrolyte multilayers.

As mentioned above, the build-up of LbL multilayers is driven by electrostatic interactions between oppositely charged constituents. This build-up can precisely control the composition, morphology and structure of the film. Briefly, the LbL self-assembly proceeds as follows (Figure 1): A charged substrate is immersed in a solution consisting of oppositely charged polyelectrolytes to form the first monolayer via absorption. This step is followed by a washing step to remove the weakly bound or unbound species. In addition, this wash step can prevent cross contamination of oppositely charged polyelectrolytes. Then, the monolayer-coated substrate is immersed in another solution of oppositely charged polyelectrolytes to form the second monolayer via absorption. This process is repeated until the desired multilayers are formed (De Villiers et al., 2011; Cui et al., 2016).

In the assembly process, electrostatic attraction is the main force. In addition, hydrogen bonding, hydrophobic interactions, covalent interactions, and biological interactions also play a role (Lvov et al., 1999; De Villiers et al., 2011; Cui et al., 2016). A variety of substances (e.g., titanium, glass, and silicon wafers) can be used as the substrate materials in the assembly without significantly altering the physical and mechanical properties of the substrate (De Villiers et al., 2011; Shukla and Almeida, 2014). In addition, the assembly process can be performed under mild conditions rather than special conditions involving high temperature and high pressure. Many polyelectrolytes can be utilized to form the multilayers, including proteins, nucleic acids, drugs, and inorganic nanoparticles (Macdonald et al., 2011; Shukla and Almeida, 2014; Castleberry et al., 2016).

Despite the self-assembly process being simple and mild, it can be affected by many factors, such as the concentration and ionic strength of the polyelectrolyte solution, pH, temperature, assembly time, molecular weight, and size. These factors will affect the morphology, thickness, and other biological characteristics of the assembled film (Elbert et al., 1999; De Villiers et al., 2011).

### Surface modification of titanium implant by LbL

To apply LbL to the titanium implant, the surface of the titanium implant should be charged first by physical or chemical methods, such as application of polyethylenimine, or dopamine conjugation. Then the PEMs were prepared by the methods mentioned above. Many substances could be applied to the implant to affect the surface properties of the implant surface. LbL can affect the surface prosperities of the titanium implants in several aspects. Based on the different biological effects of the substances, LbL modified implant could promote adhesion, proliferation and differentiation of related cells on the titanium surface and enhance the osseointegration and antibacterial properties of titanium implants. In addition, local gene therapy and sustained-release of drugs around the implant can be achieved, which has motivated research of new type dental implants. Besides, PEMs on the dental implant surface can change the hydrophilicity of the dental implants and a high degree of hydrophilicity could promote differentiation and maturation of osteoblasts, which will contribute to an acceleration of osseointegration (Zhao et al., 2005).

### Advantages of LbL modified titanium implant

**Figure 2:**
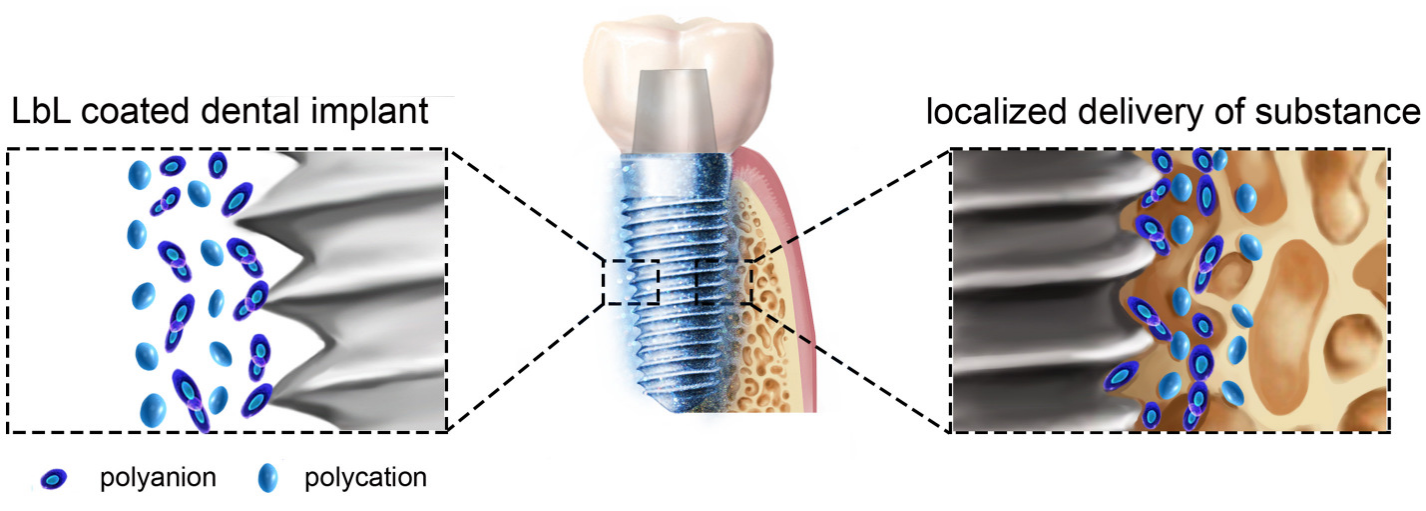
A schematic illustration of LbL self-assembly dental implants. LbL coated titanium dental implant and localized delivery of substance to its surrounding environments to provide some benefit effects.

Compared with the other methods shown in the Supplementary Table 1, LbL technique has many advantages (Becker et al., 2010; De Villiers et al., 2011; Zhong et al., 2016). (1) The assembly process is simple and mild and does not require special equipment. (2) The PEMs is formed via electrostatic interactions between molecules under mild conditions, which could maintain their biological activity. For example, some cytokines and small interfering RNA (siRNA) are easy to deactivation, while by LbL the cytokines and siRNAs can be easily transported to the surrounding of the implants to achieve their biological functions. (3) The targeted, sustained-release administration can be easily achieved by adding different substances or adjusting the physical and chemical properties of the self-assembled materials. (4) The morphology of the formed film can be controlled at the nanoscale to achieve the desired thickness, biocompatibility, and other characteristics. In general, the methods in Supplementary Table 1 are worked largely depends on the change of titanium implants surface morphology, while LbL could effectively convey many substances to play their biological effects around the implant in a simple method (Figure 2).

## Effects of LbL modified titanium implant

### Promoting osteogenesis and osseointegration

Good osseointegration is a prerequisite for the success of a dental implant. Therefore, the enhancement of osseointegration is an important research area that has been widely studied. After surface modification by LbL, titanium and its alloys promote the attachment and osteogenic differentiation of related osteoblasts or stem cells and increase the expression of related osteogenesis markers. The commonly used colloids include cytokines, DNA plasmid, etc.

#### Cytokines

Hu et al. constructed a bioactive multilayered structure consisting of a gelatin/chitosan pair that contained BMP2 and fibronectin (FN) on the Ti6Al4V surface via a LbL assembly technique (Hu et al., 2012). This BMP2/FN-containing multilayer can control the delivery of the factor and simultaneously regulate adhesion and osteogenic differentiation potency of the mesenchymal stem cells (MSCs). The BMP2 in the film exhibited sustained release *in vitro* and after 14 days, and ~16% of the BMP2 remained within the multilayered structure on the substrates. Based on cellular research, this PEMs promotes adhesion, stretch and osteogenic differentiation of bone marrow MSCs. More importantly, micro-CT analysis and histological observations demonstrated that the multilayer-coated Ti6Al4V implants promoted bone density and new bone formation. In addition, the modified implants inhibited the differentiation of RAW264.7 cells into osteoclasts. To enhance the long-term survival of titanium implants in patients with osteoporosis, calcitonin and BMP2 were self-assembled onto the surface of titanium alloys, and then, this material was implanted into rabbit osteoporosis models. This modification substantially promoted the formation and remodeling of new bone in osteoporotic rabbits and inhibited osteoclasts differentiation, indicating good anti-osteoporosis and bone inducing capacity. Overall, this multilayer coating has the potential for clinical application in patients with osteoporosis (Huang et al., 2016).

#### DNA and siRNA

The LBL method provides control over the incorporation of DNA into multilayered polyelectrolyte assemblies. Jiang et al. employed hyaluronic acid (HA) as a polyanion and liposomes containing rhBMP-2 plasmid DNA as a polycation to modify a titanium surface (Jiang et al., 2012). After assembly of 8 layers, the plasmid DNA content in the multilayer film was as high as 7.56 ± 0.68 ng/cm^2^. After culturing for 3 days, the concentration of BMP2 that was secreted by the cells reached 0.75 ng/mL. These newly synthesized PEMs promoted MC3T3-E1 cell adhesion and proliferation by significant up-regulation of the expression of osteoblast differentiation markers. However, its sustained release was weak. After 3 days, the amount of DNA was only 50% and after 7 days, only 25% of the DNA remained. In animal studies, histologic evaluation of the implants indicated that after 4 weeks of healing, the BIC for the modified implant group was much lower than that for the control surface. However, this change was not significant. In contrast, the percentage of bone within the threads of the implants was significantly higher than the control group. This result may be related to the short lasting time of BMP2 and liposomes that are toxic (Jiang et al., 2013). To explore a spatiotemporally controllable gene delivery system with high efficiency and safety, PEMs were constructed of titanium via LbL technique using plasmid deoxyribonucleic acid-loaded lipopolysaccharide–amine nanopolymersomes (pNPs) as polycations and acid (HA) as polyanions by Teng et al. The transfection efficiency of the modified PEMs reached 60.98% after 48 h, and this modified method also had good biological effects, including enhancing the proliferation and osteogenic differentiation of mesenchymal stem cells (Teng et al., 2014a,b, 2016).

siRNA technology is a promising approach for sequence-specific targeting of mRNAs for destruction, enabling the knockdown of virtually any expressed protein (Whitehead et al., 2009). Based on the characteristics of siRNA, which is negatively charged, Song et al. designed and synthesized siRNA with interference casein kinase 2 interacting protein 1 (Ckip-1). In addition, sodium hyaluronate and chitosan/siRNA (CS/siRNA) nanoparticles were employed as the polyanion and polycation, respectively, to construct a PEMs on titanium surface (Zhang L. et al., 2015; Zhang Y. et al., 2015). Ckip-1 is an intracellular negative regulator of bone formation that does not affect bone resorption, and downregulation of Ckip-1 by siCkip-1 can significantly promote osteogenesis under both normal and osteoporotic conditions. The cumulated load of siRNA increased linearly with the bilayer number, and gradual release in the film allowed siRNA to be maintained on the titanium surface for more than ~1 week with almost no cytotoxicity. The LbL film loading with osteogenic siRNA could effectively suppress Ckip-1 expression and substantially increase the osteogenic differentiation in MG63 cells. This indicates us the potential for osteoporotic application by this method.

#### Natural polymeric substances

Several studies have reported the direct modification of the titanium surface with natural polymeric substances via LbL. Li et al. employed the natural calcium binding property of casein (CA) to form a multilayer film with chitosan by via LbL technique (Li et al., 2016). The mineralization experiment in simulated body fluid demonstrated that this multilayer film promoted the formation of hydroxyapatite crystal aggregates that exhibited better *in vivo* mineralization. In addition, this multilayer film significantly promoted MSCs attachment, proliferation and osteogenic differentiation. Sakurai et al. prepared the film that was assembled with DNA and protamine and demonstrated that DNA/protamine PEMs exhibit a strong mineralization ability (Sakurai et al., 2016). The results from animal experiments indicated that PEMs-coated titanium implants provided a significantly higher bone-to-implant contact (BIC) ratio 3 weeks after implantation. However, no difference between the modified group and control group was observed 9 weeks after implantation. Therefore, this multilayered implant promotes new bone formation in the early stages of the bone healing process.

### Promoting the formation and healing of soft tissues

The success of dental implant treatment depends on the healing of hard tissues and the formation and healing of soft tissues around the implants. A good biological seal between the soft tissue and the implant may not only prevent oral bacteria and their products from penetrating the body and minimizing the risk of peri-implantitis but can also improve the aesthetics of the implant restoration (Hsu et al., 2014). Werner et al. applied poly-lysine, and poly-L-glutamic acids were alternatingly deposited to form PEMs for the use as the transmucosal part in the implants. An *in vitro* study demonstrated that this film could promote proliferation of epithelial cells, and the colonization of the microporous titanium surface was enhanced. The results from an animal study indicate that the microstructure of the implant neck combined with the LbL multilayer films provides efficient routes for improving the integration of soft tissues on titanium implants (Werner et al., 2009). Yang et al. fabricated a multilayer consisting of chitosan/laminin γ2 DNA coating on a titanium surface and evaluated its biological properties via *in vitro* study (Yang et al., 2016). When the film consisted of 5 layers, its gene transfection efficiency was as high as 20%. HEK293 cells cultured on the multilayer films exhibited a significantly higher adhesion activity than the control group. In addition, the expressions of laminin γ2, integrin β4, and net protein in HN4 cells increased. The results indicated this multilayer laminin γ2 DNA coating may be effective for improving cell adhesion and the formation of hemidesmosomes on titanium surfaces.

### Improving the antibacterial properties of titanium

Peri-implantitis and peri-implant mucositis can also lead to dental implant failure. Bacteria adhesion and subsequent biofilm formation are the primary causes of implant-associated infections (Alradha et al., 2012; Renvert and Quirynen, 2015). The biofilms could result in the bacteria becoming more intrusive and protect them from attacking the host and antibiotics (Shi et al., 2015). Therefore, the antibacterial properties of the implant must be enhanced to prevent colonization of bacterial and formation of biofilms.

#### Nano-sliver

Nano-silver is widely used as an antimicrobial agent in the oral cavity and exhibits good antibacterial effects and the ability to resist drug resistance (Qureshi et al., 2014). Zhong et al. applied the LbL technique to the fabrication of a silver nanoparticle-containing multilayer coating on titanium surfaces (Zhong et al., 2016). The results indicated that the sustained release time of nano-silver in this PEMs *in vitro* was more than 14 days, and this film exhibited good antibacterial function. The PEMs loaded with silver nanoparticles can kill 100% of the planktonic and adherent bacteria (*Staphylococcus aureus*) during first 4 days, and this antibacterial efficacy against planktonic and adherent bacteria was 65–90% after 14 days. A cellular experiment demonstrated that the nano-silver exhibited slight cytotoxicity. However, this toxicity can be reduced by adjusting the concentration and release rate of the nano-silver. Li et al. modified titanium substrates with silver nanoparticle-embedded sulfhydrylated chitosan/gelatin PEMs films to increase the antibacterial properties of titanium (Li et al., 2014). In this process, the silver ions were stored by binding to the sulfhydryl of chitosan. *In vitro* experiments demonstrated that the modified method efficiently inhibited the growth and activity of *Bacillus subtitles* and *Escherichia coli* on titanium surfaces. Moreover, *in vitro* results confirmed that titanium substrates that are coated with functional multilayer films retain the biological functions of osteoblasts.

#### Antibiotics or antibiotic peptides

Using LbL self-assembly technique, antibiotics, or antibiotic peptides can also be loaded into the PEMs to enhance the antibacterial properties of titanium. Lv et al. dissolved the minocycline in the alginate solution and modified the surface of titanium with chitosan/alginate PEMs containing minocycline prepared via the LbL technique (Lv et al., 2014). The sustained release time of minocycline *in vitro* was up to 14 days, and minocycline also had a strong ability to kill *Staphylococcus aureus*. Alexandra et al. loaded gentamicin onto a PEMs consisting of chitosan/poly-β-cyclodextrin. Antimicrobial investigations revealed that the PEMs exhibited a microbial activity for up to 6 days of incubation with *S. aureus* (Pérez-Anes et al., 2015). After linking a broad spectrum antimicrobial peptide (AMP) Tet213 with collagen IV (renamed as AMPCol), Shi et al. modified titanium substrates with AMPCol and HA via an LbL technique to increase the antibacterial properties. This coating with controlled release of AMP decreased the growth of both *Staphylococcus aureus* and *Porphyromonas gingivalis* for up to 1 month and inhibited early biofilm formation. In addition, these PEMs encouraged cellular attachment with low levels of cytotoxicity or erythrocyte hemolysis (Shi et al., 2015).

### Local drug delivery and sustained release

LbL self-assembly has been widely used to generate controlled and sustained drug release PEMs because it allows for desired functions and structures to be obtained through a simple procedure. To enhance the osseointegration of dental implants in diabetic or osteoporotic patients, researchers have attempted to incorporate related drugs into the PEMs to modify the titanium surface. Xu et al. prepared various layers of a heparin/chitosan coating via LbL technique and loaded the drug HU-308 by physical adsorption (Qian et al., 2014). The sustained release time of the anti-osteoporosis drug (HU-308) *in vitro* was more than 30 days. In osteoporotic rat models, HU-308 coated on titanium implants promoted new bone formation and bone mineralization in the early to middle stage of implantation and improved osseointegration at the implant-bone interface. This type of implant may provide a new possibility for promoting implant–bone osseointegration for osteoporotic patients.

## Summary and outlook

Improving the biocompatibility of the implant, achieving good osseointegration between implants and surrounding bone tissues, forming good soft tissue closure with the surrounding soft tissues, reducing infections around the implant and improving the success rate of implants are important factors in the field of oral implantology. The LbL self-assembly technique provides a new approach for achieving these goals. As mentioned above, LbL technique has been widely used for the modification of titanium and its alloys with good results, which provide the foundation for further clinical application.

However, some problems still need to be solved. First, the sustained release time of PEMs must be improved. Currently, the sustained release time in most studies is ~14 days with some times that are even shorter. Second, the cell transfection efficiency is low for assembled nucleic acids. Therefore, additional study is required to achieve higher transfection efficiency and enhanced biological effects. Third, few research focused on bonding strength between the implant and the PEMs. Finally, most of the current studies are performed *in vitro*, and *in vivo* studies have been limited. Therefore, additional studies are needed to solve these problems, and we believe that there will be a breakthrough in the near future to further shorten the treatment time, reduce complications and improve the success rate of dental implants.

## Author contributions

QS and ZQ summarized the literature and wrote the manuscript. DL prepared figures and modified the manuscript. HL supervised all the works and wrote the manuscript.

### Conflict of interest statement

The authors declare that the research was conducted in the absence of any commercial or financial relationships that could be construed as a potential conflict of interest.
