# Supplementary material for: Surface Modification of Dental Titanium Implant by Layer-by-Layer Electrostatic Self-Assembly
Source: Front Physiol. 2017 Aug 7;8:574. doi: 10.3389/fphys.2017.00574 (PMC5545601; doi:10.3389/fphys.2017.00574)
Supplement: Supplementary file 1 [file Table1.DOCX]

**Supplementary Table 1. Techniques used for surface modification of titanium implant.**

| **Category** | **Method** | **Reference** |
| --- | --- | --- |
| Subtractive methods | Grit blasting | ([Shemtov-Yona et al., 2014](#_ENREF_8)) |
|  | Acid etching | ([Chiang et al., 2016](#_ENREF_1)) |
|  | Laser ablation | ([Nevins et al., 2010](#_ENREF_5)) |
|  | Alkali-heat treatment  ( sodium hydroxide) | ([Xing et al., 2014](#_ENREF_10)) |
| Additive methods  (surface coating) | Plasma spraying | ([Kato et al., 2005](#_ENREF_3)) |
|  | Monolayer self-assembled | ([Tack et al., 2015](#_ENREF_9)) |
|  | Layer-by-Layer self-assemble | ([Yang et al., 2016](#_ENREF_11)) |
|  | Ion beam treatment | ([Maitz et al., 2002](#_ENREF_4)) |
| Subtractive/Additive methods | Anodization | ([El-wassefy et al., 2014](#_ENREF_2)) |
|  | Micro-arc oxidation | ([Ribeiro et al., 2015](#_ENREF_7)) |
| others | Photofunctionalization (UV treatment) | ([Park et al., 2013](#_ENREF_6)) |

**Reference**

Chiang, H.J., Hsu, H.J., Peng, P.W., Wu, C.Z., Ou, K.L., Cheng, H.Y., Walinski, C.J., and Sugiatno, E. (2016). Early bone response to machined, sandblasting acid etching (SLA) and novel surface-functionalization (SLAffinity) titanium implants: characterization, biomechanical analysis and histological evaluation in pigs. *J Biomed Mater Res A* 104**,** 397-405.

El-Wassefy, N.A., Hammouda, I.M., Habib, A.N., El-Awady, G.Y., and Marzook, H.A. (2014). Assessment of anodized titanium implants bioactivity. *Clin Oral Implants Res* 25**,** e1-9.

Kato, R., Nakamura, S., Katayama, K., and Yamashita, K. (2005). Electrical polarization of plasma-spray-hydroxyapatite coatings for improvement of osteoconduction of implants. *J Biomed Mater Res A* 74**,** 652-658.

Maitz, M.F., Pham, M.T., Matz, W., Reuther, H., Steiner, G., and Richter, E. (2002). Ion beam treatment of titanium surfaces for enhancing deposition of hydroxyapatite from solution. *Biomol Eng* 19**,** 269-272.

Nevins, M., Kim, D.M., Jun, S.H., Guze, K., Schupbach, P., and Nevins, M.L. (2010). Histologic evidence of a connective tissue attachment to laser microgrooved abutments: a canine study. *Int J Periodontics Restorative Dent* 30**,** 245-255.

Park, K.H., Koak, J.Y., Kim, S.K., Han, C.H., and Heo, S.J. (2013). The effect of ultraviolet-C irradiation via a bactericidal ultraviolet sterilizer on an anodized titanium implant: a study in rabbits. *Int J Oral Maxillofac Implants* 28**,** 57-66.

Ribeiro, A.R., Oliveira, F., Boldrini, L.C., Leite, P.E., Falagan-Lotsch, P., Linhares, A.B., Zambuzzi, W.F., Fragneaud, B., Campos, A.P., Gouvea, C.P., Archanjo, B.S., Achete, C.A., Marcantonio, E., Jr., Rocha, L.A., and Granjeiro, J.M. (2015). Micro-arc oxidation as a tool to develop multifunctional calcium-rich surfaces for dental implant applications. *Mater Sci Eng C Mater Biol Appl* 54**,** 196-206.

Shemtov-Yona, K., Rittel, D., and Dorogoy, A. (2014). Mechanical assessment of grit blasting surface treatments of dental implants. *J Mech Behav Biomed Mater* 39**,** 375-390.

Tack, L., Schickle, K., Boke, F., and Fischer, H. (2015). Immobilization of specific proteins to titanium surface using self-assembled monolayer technique. *Dent Mater* 31**,** 1169-1179.

Xing, H., Komasa, S., Taguchi, Y., Sekino, T., and Okazaki, J. (2014). Osteogenic activity of titanium surfaces with nanonetwork structures. *Int J Nanomedicine* 9**,** 1741-1755.

Yang, G., Zhang, J., Dong, W., Liu, L., Shi, J., and Wang, H. (2016). Fabrication, characterization, and biological assessment of multilayer laminin gamma2 DNA coatings on titanium surfaces. *Sci Rep* 6**,** 23423.
